# On the Diagnosis of Syphilis

**Published:** 1804-12-01

**Authors:** 


### *To the Editors of the Medical and Phyfical Journal.*

GENTLEMEN,

As the following case appears to be strongly illustrative of the observations lately published by Mr. Abernethy, on diseases resembling syphilis, the importance of which must be obvious to every one; I have thought it worthy of publication. If you are of the same opinion, you will, by inserting it in your valuable Miscellany, oblige,

A CONSTANT READER.

---

J. H. a young man of a sanguineous temperament, and apparently of a scrophulous habit, applied to me the latter end of February last, for the cure of a venereal sore throat. On enquiring into his case, he assured me that he had had no primary symptoms whatever. He informed me, however, that, about ten months previous to this attack, he had undergone a mercurial course for the cure of a suppurating buboe and sore throat, which had been preceded by chancres. At the time the mercury was left off, the buboe had not healed, having degenerated into a fistulous sore, which, however, had quickly healed on being laid open, and from that time he had remained perfectly free from every symptom of syphilis till he had occasion to apply to me. On a stricter enquiry as to the symptoms preceding the affection of his throat, of which he now complained, I found that he had felt a slight degree of irritation about the glans penis a few days after coition, attended with a very slight discharge from the orifice of the urethra, which ceased almost immediately on washing the parts with cold water. This was succeeded by some degree of stiffness and uneasiness in the groin, which had been before affected, and in which a little fulness and hardness had been perceptible ever since the healing of the sinus. This affection of the groin, however, was so slight, as not to give him any alarm; and there being not the slightest appearance of chancre, he did not apply to me till his throat became sore; on looking into which, I discovered an ulcer on the right tonsil, which had all the characteristics of a venereal sore, viz. a circumscribed hollow sore, the bottom covered with a white slough, and the surrounding edges much inflamed. The situation of my patient patient being such, that he could not, without the greatest inconvenience, rub in mercury, I began by giving him hydr. muriat. gr. ⅛ ter quotidiè. Under this treatment the ulcer in a few days ceased to spread, and in little more than a fortnight was completely healed. But being unwilling to trust to this remedy alone, and also conceiving that, since he had no primary symptom, this affection of his throat might have been owing to some syphilitic virus, which had long lain dormant in the system, till called into action by the irritation of the glands abovementioned, I strongly recommended to my patient a further continuance of the use of mercury, to which, having suffered but slightly from it, he readily consented. I therefore gave him the pil. hydrarg. in such doses as to keep the gums slightly affected for five weeks longer. Having now undergone a two months course of mercury, I thought he might safely leave it off, and advised him accordingly. But within forty-eight hours after this, he began to complain of his throat again, which appeared considerably inflamed. Attributing this to an improper exposure to cold, while still under the influence of mercury, I contented myself with giving him a gargle, and desiring him to keep his throat warm. In a few days he again applied to me, and told me he thought there was an ulcer in his throat; and, on examination, I discovered one on the left tonsil, less circumscribed, and more superficial, than the one which had appeared on the right tonsil, but still having the white slough at bottom, and the inflamed edges characteristic of a venereal sore. I had again recourse to the hydrarg. muriat. which not appearing to produce any effect upon the sore, I gave the pil. hydrarg. so as to affect the mouth considerably. Still, however, the ulcer shewed no symptoms of amendment, nor did it spread; but above it two other ulcers broke out, about the size of a small pea each, but without the venereal character. I now began to have some doubts as to the nature of this affection, and had almost resolved to leave off the mercury, and put the patient upon a course of sarsaparilla, &c. when, as the mouth became more affected, the smaller ulcers healed. The original one, however, continued nearly in statu quo; and, at the same time, a great deal of scurf began to collect about the roots of the hair. I therefore continued the mercury some days longer, when I happened to meet with Mr. Abernethy's valuable work. The perusal of this, determined me to leave off the use of mercury immediately, and trust to nature for the removal of these

 apparently apparently syphilitic symptoms. For near a month the ulcer in the throat underwent no alteration; and, during this time, two small ulcers, similar to the ones above described, broke out, and healed again in a few days. It is now upwards of two months since the mercury was left off, his throat is quite well, the roots of his hair entirely free from scurf, and he has shewn no other symptom whatever of any syphilitic taint remaining in the habit.

The above case suggests some very curious enquiries. Was the ulcer, which first appeared in the throat, venereal or not? If it was, how did it arise? Is it possible for the venereal poison to be absorbed into the system so as to produce secondary disease, without having first excited local disease, as chancre or bubo? Or, might the ulcer have arisen from virus, which had lain dormant in the hardened glands since the healing of the sinus in the groin, which the stimulus produced upon the glans penis by coition, and communicated to the absorbents of the groin, had excited them to take up into the system? Or, did all these symptoms arise from acrimonious matter, not venereal, as supposed by Mr. Abernethy, communicated during coition? There is every reason to conclude, although the short space of time elapsed since the mercury was left off will not allow complete certainty upon this head, that the ulcers which appeared in the second instance were not venereal; and hence we may be led to doubt, whether the first affection of the throat was so, although it so readily disappeared under the use of mercury. These are enquiries of great importance, and deserve the attentive consideration of those who are more equal to the task, than the author of these observations.

*Sept.* 6, 1804.

